# A DEL-1/αvβ3 integrin axis promotes brown adipocyte progenitor proliferation and cold-induced brown adipose tissue adaptation

**DOI:** 10.1016/j.molmet.2025.102229

**Published:** 2025-08-07

**Authors:** Kyoung-Jin Chung, Antonios Chatzigeorgiou, Jong-Hyung Lim, Xiaofei Li, Ismini Marava, Dong-Young Kim, Anke Witt, Janine Gebler, Sylvia Grossklaus, Bettina Gercken, Irakli Kopaliani, Pallavi Subramanian, Matthias Blüher, Khalil Bdeir, Vasileia Ismini Alexaki, George Hajishengallis, Triantafyllos Chavakis

**Affiliations:** 1 Institute for Clinical Chemistry and Laboratory Medicine, University Hospital and Faculty of Medicine, Technische Universität Dresden, Dresden, Germany; 2 Department of Physiology, Medical School, National and Kapodistrian University of Athens, Athens, Greece; 3 Department of Basic and Translational Sciences, Laboratory of Innate Immunity and Inflammation, Penn Dental Medicine, University of Pennsylvania, Philadelphia, PA, USA; 4 Sheng Yushou Center of Cell Biology and Immunology, School of Life Sciences and Biotechnology, Shanghai Jiao Tong University, Shanghai, China; 5 Institute of Physiology, Faculty of Medicine, Technische Universität Dresden, Dresden, Germany; 6 Helmholtz Institute for Metabolic, Obesity and Vascular Research (HI-MAG) of the Helmholtz Center Munich at the University of Leipzig and University Hospital Leipzig, Leipzig, Germany; 7 German Center for Diabetes Research, Neuherberg, Germany; 8 Department of Pathology and Laboratory Medicine, Perelman School of Medicine, University of Pennsylvania, Philadelphia, PA, USA; 9 Paul Langerhans Institute Dresden, Helmholtz Center Munich, University Hospital and Faculty of Medicine, Technische Universität Dresden, Dresden, Germany

**Keywords:** Developmental endothelial locus-1, Brown adipose tissue, Integrins, Brown adipocyte progenitor proliferation, Cold-induced brown adipose tissue adaptation, Thermogenesis

## Abstract

**Objectives:**

Cold-triggered adaptation of the brown adipose tissue (BAT) promotes increased non-shivering thermogenesis and helps maintain body temperature. This study investigated the role of the secreted protein developmental endothelial locus-1 (DEL-1) in regulating BAT adaptation to cold.

**Methods:**

DEL-1 expression in BAT was assessed following cold exposure in mice. The role of DEL-1 in cold-induced BAT adaptation, thermogenesis and proliferation of brown adipocyte progenitor cells was analyzed by utilizing genetically modified mouse models. Mechanistic insights into DEL-1-mediated regulation of brown adipocyte progenitor proliferation were obtained through in vitro assays.

**Results:**

DEL-1 was expressed in the vascular endothelium of the BAT and its expression was upregulated upon cold exposure. By interacting with αvβ3 integrin on brown adipocyte progenitor cells, DEL-1 promoted their proliferation in a manner dependent on AKT signaling and glycolysis activation. Compared to DEL-1-sufficient mice, DEL-1-deficient mice or mice expressing a non-integrin-binding mutant of DEL-1 carrying an Asp-to-Glu substitution in its RGD motif, displayed decreased cold tolerance. This phenotype was associated with impaired BAT adaptation to cold and reduced brown adipocyte progenitor cell proliferation. Conversely, endothelial-specific DEL-1 overexpression in DEL-1-deficient mice restored the BAT thermogenic response to cold.

**Conclusions:**

Together, the DEL-1/αvβ3 integrin-dependent endothelial-brown adipocyte progenitor cell crosstalk promotes cold-stimulated BAT adaptation. This knowledge could be potentially harnessed therapeutically for promoting BAT expansion towards improving systemic metabolism.

## Highlights

• Cold stress upregulates endothelial DEL-1 expression in the brown adipose tissue (BAT).
• DEL-1 enhances brown adipocyte progenitor cell (APC) proliferation via interaction with αvβ3 integrin.
• The DEL-1/αvβ3 integrin-dependent endothelial cell-APC crosstalk promotes cold-induced BAT adaptation.

## 1 Introduction

The brown adipose tissue (BAT) is the main site for non-shivering thermogenesis in rodents and human neonates and helps maintain body temperature [1,2]. Brown adipocytes display multilocular lipid droplets and are rich in mitochondria; moreover, expression of uncoupling protein 1 (UCP-1) allows them to generate heat by dissociating mitochondria function from ATP production, as UCP-1 dissipates the proton gradient at the inner mitochondrial membrane [[2], [3], [4]]. Furthermore, cells with brown adipocyte-like properties have been described in the white adipose tissue and defined as beige adipocytes; their emergence can be triggered by cold exposure [2,4]. In mice, BAT is predominantly located in the interscapular region [2]. Active brown/beige adipose tissue was discovered in adult humans as well [5,6]. Due to their substantial capacity for energy dissipation, brown and beige adipocytes play an important role not only in body temperature regulation but also in systemic metabolism, including glucose and lipid homeostasis [4,7,8]. Recent research findings in fact suggest that improving the function of brown and beige adipocytes may promote metabolic health and may serve as a potential therapeutic approach in type 2 diabetes and cardiometabolic disease [4,[9], [10], [11], [12]].

Upon cold exposure, the BAT rapidly increases its thermogenic capacity, which is driven by several complementary mechanisms, largely orchestrated by sympathoadrenergic stimulation [2,13]. For instance, the cold-induced thermogenic recruitment process of BAT includes upregulation of UCP-1 expression, as well as activation of the mTOR pathway and a consequent increase in glucose uptake and glycolysis of BAT [2,[14], [15], [16]]. Importantly, the BAT displays an enormous capacity to rapidly expand upon cold exposure [13]. Cold-induced BAT adaptation is linked with a rapid (within hours) and massive increase in proliferation of different interstitial cell types in the BAT [13]. Proliferating cells include endothelial cells, thereby resulting in enhanced angiogenesis of the expanding BAT, and perivascularly located stromal cells [13,17,18]. The latter population harbors the platelet-derived growth factor receptor alpha (PDGFRα)-expressing adipocyte progenitor cells (APC); these cells derive from Myf5^+^ precursors and give rise to new brown adipocytes [[18], [19], [20], [21]]. In the white adipose tissue, besides enhanced APC growth, cold-induced beige adipogenesis also involves the phenotypic transdifferentiation of mature white adipocytes to beige cells with enhanced thermogenic capacity [[22], [23], [24]]. Noteworthy, despite the rapid concomitant cold-induced activation of spatially neighboring endothelial cells and APCs in the BAT, very little information exists about the potential crosstalk of these cell types during BAT cold acclimation.

Developmental endothelial locus-1 (DEL-1), also designated EDIL3 (EGF-like repeats and discoidin I-like domains 3), is a secreted protein that associates with the extracellular matrix and is produced by endothelial and stromal cells [[25], [26], [27]]. DEL-1 is capable of interacting with different integrin receptors, including integrins that recognize the Arg-Gly-Asp (RGD) motif, such as αvβ3 integrin, due to the presence of this motif in its second EGF-like repeat [25,28,29]. DEL-1 is a locally acting anti-inflammatory factor that regulates tissue immune plasticity [25,26,[29], [30], [31], [32], [33], [34], [35], [36]]. DEL-1 plays an important role in promoting tissue homeostasis and repair, as exemplified particularly in the context of hematopoiesis and bone regeneration, a function that is attributed to its interaction with β3 integrin on respective hematopoietic or osteoprogenitor cells [27,37,38]. For instance, DEL-1 stimulates a β3 integrin- and focal adhesion kinase (FAK)-dependent pathway in osteoprogenitor cells driving bone regeneration [38]. Interestingly, αvβ5 integrin, which is closely related to αvβ3 integrin, was recently shown to promote both bone remodeling and cold-stimulated beige fat neogenesis, via interaction with irisin, a cleavage product of fibronectin type III domain-containing protein 5 (FNDC5), in a manner that also involved FAK signaling [24,39]. These aspects prompted us to address the role of DEL-1 and its interaction with αvβ3 integrin in the crosstalk between endothelial cells and APCs in cold-induced BAT activation. We found that cold exposure enhanced endothelial DEL-1 expression in the BAT; endothelial DEL-1 promoted cold-induced BAT adaptation, in a manner that involved glycolysis upregulation and β3 integrin-dependent APC proliferation. Our findings define the DEL-1/αvβ3 integrin interaction as a novel juxtacrine mechanism in cold-induced BAT adaptation.

## 2 Results

### 2.1 Endothelial DEL-1 upregulation in BAT following cold exposure

**Figure 1:**
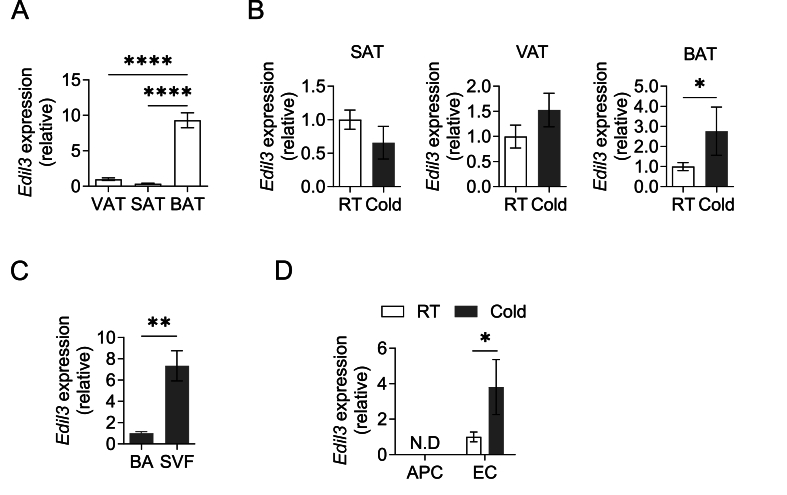
**DEL-1 expression in adipose tissue depots.** (A) *Edil3* mRNA expression in visceral, subcutaneous and brown adipose tissue depots of wild-type mice (VAT, SAT and BAT, respectively). 18S expression was used for normalization and *Edil3* expression of VAT was set as 1 (*n* = 6 mice). (B–D) Wild-type mice were challenged with a temperature of 4 °C (Cold) or remained at room temperature (RT) for 12 h. (B) *Edil3* mRNA expression in the SAT, VAT and BAT (*n* = 4–5 mice per group). 18S expression was used for normalization and *Edil3* expression under RT conditions for each tissue was set as 1. (C) *Edil3* mRNA expression in the adipocyte fraction containing brown adipocytes (BA) and in stromal vascular fraction (SVF) cells from the BAT upon cold exposure (*n* = 4 mice). 18S expression was used for normalization and *Edil3* expression of BA was set as 1. (D) *Edil3* mRNA expression in adipocyte progenitor cells (APC, defined as CD45^−^CD31^−^CD29^+^Sca1^+^PDGFRα^+^ cells) and endothelial cells (EC, defined as CD45^−^CD31^+^ cells) isolated from the BAT at room temperature (RT) and upon cold exposure (*n* = 5–8 mice per group). *Actb* expression was used for normalization and *Edil3* expression of the endothelial cells under RT conditions was set as 1. N.D. (not detected). Data are mean ± SEM. ∗*P* < 0.05, ∗∗*P* < 0.01, ∗∗∗∗*P* < 0.0001. One-way ANOVA in (A), Student’s t-test in (B, C) except for BAT in panel (B) (Mann–Whitney *U*-test). Mann–Whitney *U*-test in (D). VAT: visceral adipose tissue, SAT: subcutaneous adipose tissue, BAT: interscapular brown adipose tissue.

We first assessed mRNA expression of the DEL-1-encoding *Edil3* gene in different adipose tissue depots of wild-type (WT) mice. We observed much higher expression of *Edil3* in the interscapular BAT than in visceral adipose tissue (VAT) or subcutaneous adipose tissue (SAT) (Figure 1A). Importantly, *Edil3* expression was further upregulated significantly in the BAT of mice upon cold challenge (4 °C) for 12 h as compared to mice kept at room temperature (Figure 1B). On the contrary, SAT and VAT mRNA expression of *Edil3* was not affected by cold exposure (Figure 1B). To identify the cellular source of the cold-triggered increased *Edil3* expression in BAT, we compared mRNA expression of *Edil3* in the adipocyte fraction harboring mature brown adipocytes (BA) and in the stromal vascular fraction (SVF) isolated from the BAT of cold-exposed mice, and found that *Edil3* expression was substantially higher in the SVF relative to the adipocyte fraction (Figure 1C). Additionally, *Edil3* expression was higher in the SVF than the adipocyte fraction in both BAT and SAT from mice kept at room temperature (Supp. Fig. 1A and B). We subsequently assessed expression of *Edil3* in isolated CD45^−^CD31^−^CD29^+^Sca1^+^PDGFRα^+^ APCs and CD45^−^CD31^+^ endothelial cells in the BAT SVF of cold-exposed and control mice (kept at room temperature). While *Edil3* expression was not-detectable in APCs, *Edil3* was expressed in the BAT endothelium and its expression was significantly increased by the cold challenge (Figure 1D). Together, expression of *Edil3* in the BAT is higher than in other fat depots and is further enhanced by cold exposure, which is due to an upregulation of DEL-1 in BAT endothelial cells.

We next analyzed expression of *Edil3* upon administration of the β3 adrenergic receptor agonist CL316243 that can also drive BAT thermogenic adaptation [40]. This treatment failed to upregulate *Edil3* expression in the BAT (Supp. Fig. 2A). Besides sympathoadrenergic activity, further mechanisms mediate the BAT adaptation to cold. For instance, thyroid hormones also play a well-established role in the cold adaptation of BAT [41]. We therefore isolated endothelial cells from the mouse BAT and treated them with 3,3′,5-Triiodo-l-thyronine (T3) or l-Norepinephrine hydrochloride (NE). Consistent with the absence of upregulation of DEL-1 expression in the BAT by the CL316243 treatment, NE did not increase *Edil3* expression in BAT endothelium. In contrast, T3 treatment significantly upregulated endothelial *Edil3* expression (Supp. Fig. 2B). Hence, the upregulation of DEL-1 in the BAT upon cold exposure does not seem to be mediated by sympathoadrenergic activity but rather by alternative mechanisms, such as the actions of thyroid hormones.

### 2.2 Endothelial DEL-1 promotes cold-induced BAT adaptation and APC proliferation

**Figure 2:**
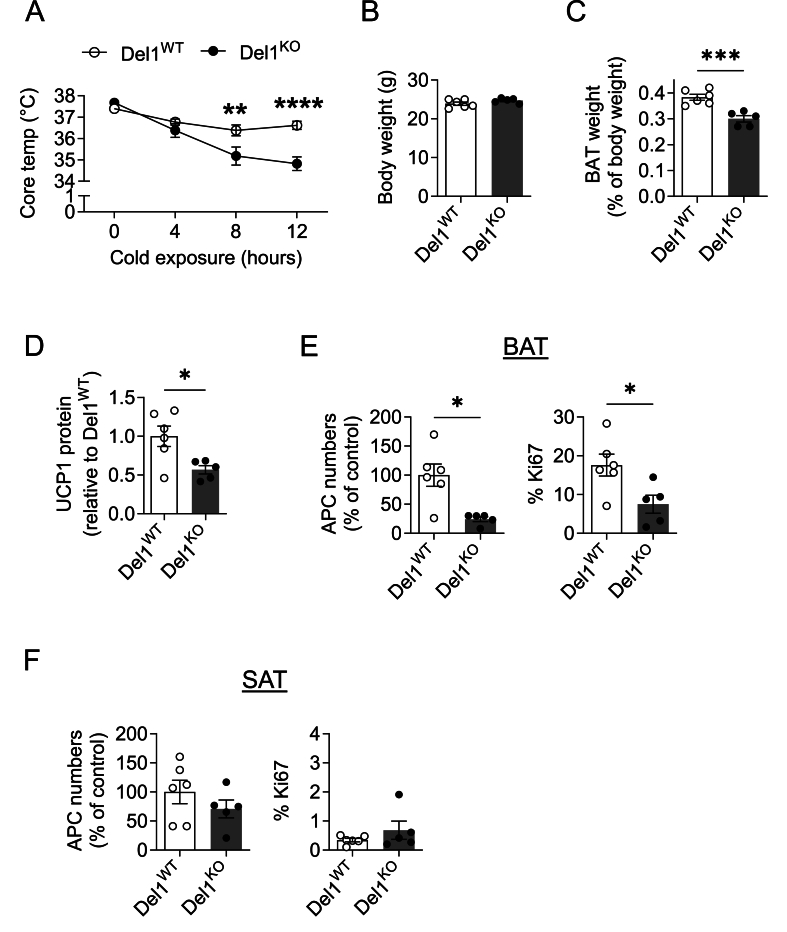
**DEL-1 promotes BAT adaptation and APC proliferation upon cold exposure**. (A–F) DEL-1-deficient (Del1^KO^, *n* = 5) and DEL-1-sufficient (Del1^WT^, *n* = 6) mice were fed a normal diet for 8 weeks as described in the Materials and Methods and then challenged with a cold exposure (temperature of 4 °C for 12 h). (A) Core body temperature during the cold exposure. (B) Body weight and (C) interscapular BAT weight as percentage of body weight of Del1^WT^ and Del1^KO^ mice after the cold challenge. (D) Protein levels of UCP-1 (pg) were analyzed by ELISA in BAT lysate samples (containing 1 μg protein) of Del1^WT^ and Del1^KO^ mice. Data are shown relative to the UCP-1 protein level of BAT from Del1^WT^ mice, set as 1. (E) The number of APCs (defined as CD45^−^CD31^−^CD29^+^Sca1^+^PDGFRα^+^ cells) (left) and the percentage of Ki67-expressing APCs (right) in the BAT of Del1^WT^ and Del1^KO^ mice after cold exposure were analyzed by flow cytometry. (F) The number of APCs (defined as CD45^−^CD31^−^CD29^+^Sca1^+^PDGFRα^+^ cells) (left) and the percentage of Ki67-expressing APCs in the SAT of Del1^WT^ and Del1^KO^ mice after cold exposure were analyzed by flow cytometry. In (E and F), the number of APCs per gram of tissue was analyzed; data are expressed as % of control (data of the Del1^WT^ mice were set as 100%). Data are mean ± SEM. ∗*P* < 0.05, ∗∗*P* < 0.01, ∗∗∗*P* < 0.001, ∗∗∗∗*P* < 0.0001. Two-way ANOVA in (A), Student’s t-test in (B–F) except for APC numbers in panel (E, left) and % Ki67 in panel (F, right), in which cases Mann–Whitney *U*-test was used.

Given the cold-stimulated upregulation of *Edil3* expression in the BAT, we next investigated if DEL-1 contributes to BAT adaptation to cold. To this end, we exposed DEL-1-deficient mice (designated thereafter Del1^KO^) [28] and DEL-1-sufficient mice (Del1^WT^) to an acute cold challenge (4 °C). Del1^KO^ mice displayed impaired cold tolerance, as revealed by a significant drop in their body temperature, related to the Del1^WT^ mice, after 8 and 12 h of cold exposure (Figure 2A). In addition, while the body weights of Del1^WT^ and Del1^KO^ mice were comparable upon cold challenge, the interscapular BAT weight (expressed as percentage of total body weight) was significantly decreased in the absence of DEL-1 (Figure 2B,C). The interscapular BAT weight of Del1^WT^ and Del1^KO^ mice did not differ at room temperature conditions (not shown). We then assessed the effect of DEL-1 deficiency on the cold-induced enhanced BAT thermogenesis by quantifying the protein expression of UCP-1. UCP-1 protein was significantly lower in the BAT of cold-exposed Del1^KO^ mice relative to that of Del1^WT^ mice (Figure 2D). Immunohistochemistry analysis for UCP-1 also revealed reduced UCP-1 staining in the BAT due to DEL-1 deficiency upon cold challenge (Supp. Fig. 3A).

Cold-induced BAT expansion and thermogenic recruitment is linked to a strong increase in cell proliferation of SVF-associated cells, particularly APCs [13,18,19]. The BAT of cold-exposed Del1^KO^ mice displayed significantly decreased APC numbers relative to the BAT of Del1^WT^ mice, which was accompanied by decreased proliferation of these cells, as assessed by Ki67 analysis (Figure 2E). The reduced APC proliferation in the BAT following cold exposure due to DEL-1 deficiency was further corroborated by the decreased mRNA expression of cell cycle-related genes *Ccna2*, *Ccnd2*, *Ccng1* in DEL-1 deficiency (Supp. Fig. 3B). As cold exposure also triggers browning in the white AT [24,42], we assessed the number of CD45^−^CD31^−^CD29^+^Sca1^+^PDGFRα^+^ APCs and their proliferation by Ki67 in the SAT of cold challenged Del1^WT^ and Del1^KO^ mice. Consistent with the lower constitutive expression of *Edil3* in the SAT compared to the BAT and the absence of *Edil3* upregulation in the SAT following cold exposure, no alterations in APC numbers and proliferation in the SAT upon cold exposure were observed due to DEL-1 deficiency (Figure 2F). Together, cold-induced BAT adaptation, including APC proliferation, failed in DEL-1 deficiency, leading to hypothermia in Del1^KO^ mice.

**Figure 3:**
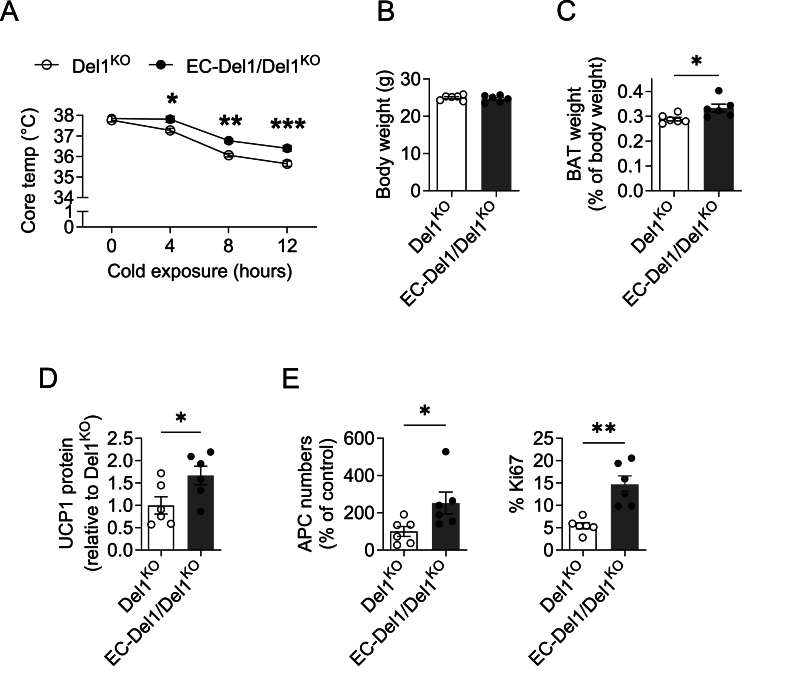
**Endothelial DEL-1 promotes cold-induced BAT adaptation and APC proliferation**. (A–E) Del1^KO^ mice (*n* = 6) and EC-Del1/Del1^KO^ mice (*n* = 6) were fed with a normal diet for 8 weeks as described in the Materials and Methods and then challenged with a cold exposure (temperature of 4 °C for 12 h). (A) Core body temperature during the cold exposure. (B) Body weight and (C) interscapular BAT weight as percentage of body weight of the Del1^KO^ and EC-Del1/Del1^KO^ mice after the cold exposure. (D) Protein levels of UCP-1 (pg) were analyzed by ELISA in BAT lysate samples (containing 1 μg protein) of Del1^KO^ and EC-Del1/Del1^KO^ mice. Data are shown relative to the UCP-1 protein level of BAT from Del1^KO^ mice, set as 1. (E) The number of APCs (defined as CD45^−^CD31^−^CD29^+^Sca1^+^PDGFRα^+^ cells) (left) and the percentage of Ki67-expressing APCs (right) in the BAT of Del1^KO^ and EC-Del1/Del1^KO^ mice after cold exposure were analyzed by flow cytometry. The number of APCs per gram of BAT was analyzed; data are expressed as % of control (data of the Del1^KO^ mice were set as 100%). Data are mean ± SEM. ∗*P* < 0.05, ∗∗*P* < 0.01, ∗∗∗*P* < 0.001. Two-way ANOVA in (A), Student’s t-test in (B–E) except for APC numbers in panel (E, left), in which case Mann–Whitney *U*-test was used.

To provide further proof that it is indeed endothelial-derived DEL-1 promoting the cold-induced BAT adaptation, we utilized our previously described endothelium-specific DEL-1 overexpressing mice (EC-Del1) [31,43,44] and bred them with DEL-1-deficient mice, thereby generating mice with exclusive endothelial-specific overexpression of DEL-1 and lacking DEL-1 in all other cell types (EC-Del1/Del1^KO^). Cold-exposed EC-Del1/Del1^KO^ mice displayed significantly higher *Edil3* expression in their BAT compared to that of WT mice (Supp. Fig. 4A). Endothelial-specific DEL-1 overexpression reversed the impaired cold tolerance of Del1^KO^ mice (Figure 3A and Supp. Fig. 4B), and restored the reduced interscapular BAT weight of Del1^KO^ mice, whereas the total body weights of cold-exposed EC-Del1/Del1^KO^ and Del1^KO^ mice were comparable (Figure 3B,C). The restored BAT thermogenic capacity of EC-Del1/Del1^KO^ mice was further confirmed by significantly increased UCP-1 protein expression in their BAT as compared to that of Del1^KO^ mice (Figure 3D). Furthermore, cold-exposed EC-Del1/Del1^KO^ mice had significantly higher BAT APC numbers and APC proliferative activity than Del1^KO^ mice (Figure 3E).

Taken together, our findings suggest that the cold-stimulated enhanced endothelial expression of DEL-1 in BAT contributes to the BAT adaptation, including increased APC proliferation, and thereby helps mice maintain their body temperature upon cold challenge.

### 2.3 DEL-1 promotes APC proliferation via interaction with αvβ3 integrin

**Figure 4:**
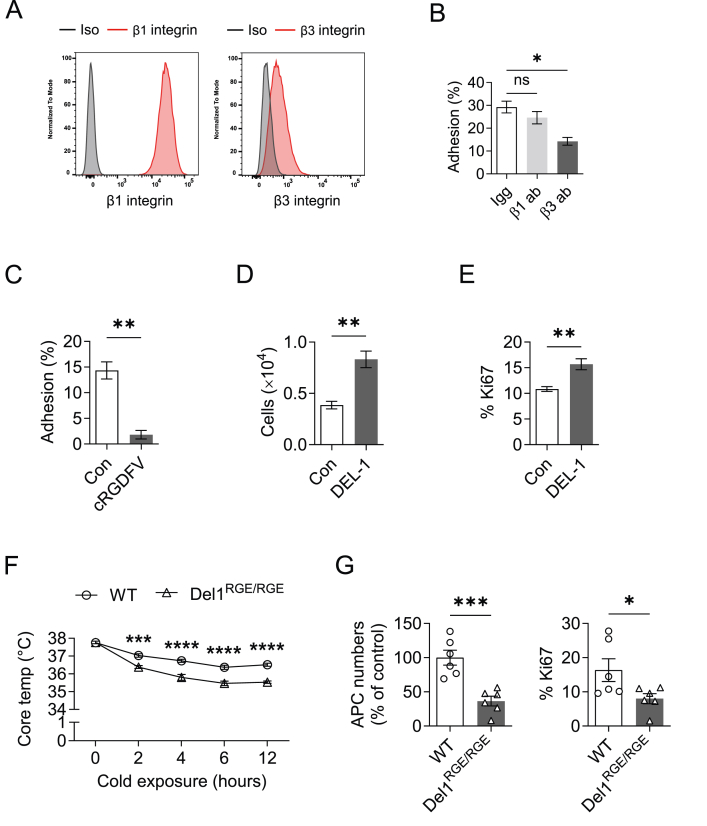
**DEL-1 promotes APC proliferation via αvβ3 integrin**. (A–E) Primary APCs were isolated by FACS sorting as CD45^−^CD31^−^CD29^+^Sca1^+^PDGFRα^+^ cells from the BAT of wild-type mice, as described under “Isolation, culture and proliferation of primary adipocyte progenitor cells (APCs) from the brown adipose tissue (BAT)” in the Materials and Methods. (A) Expression of different β integrin chains on primary APCs from the BAT was analyzed by flow cytometry; representative histograms are shown from three separate cell isolations (Iso: respective isotype control antibody). (B, C) Adhesion of APCs to immobilized recombinant DEL-1 in the presence of blocking antibody against β1 integrin (β1 ab) or against β3 integrin (β3 ab) or control antibody (Igg) (B) or in the absence (Con) or presence of RGD containing peptide antagonist with specificity for αvβ3 integrin (cRGDFV) (C). Data are shown as the percentage of adherent cells (*n* = 3 independent experiments). (D, E) Proliferation of primary APCs was studied in the absence (Con) or presence of DEL-1. Data are presented as the number of cells after 3 days of culture (the same cell number/well was seeded at the start of the assay, as described in the Materials and Methods) (D), and percentage of Ki67-expressing cells after 2 days of culture (E) (*n* = 4 independent experiments). (F, G) Del1^RGE/RGE^ mice that express an RGE point mutant isoform of DEL-1, making it incapable of interacting with αvβ3 integrin, and controls (WT) (*n* = 6 mice/group) were fed a normal diet for 8 weeks as described in the Materials and Methods and then challenged with a cold exposure (temperature of 4 °C for 12 h). (F) Core body temperature during the cold exposure. (G) The number of APCs (left) and the percentage of Ki67-expressing APCs (right) in the BAT of WT and Del1^RGE/RGE^ mice after cold exposure were analyzed by flow cytometry. The number of APCs per gram of BAT was analyzed; data are expressed as % of control (data of the WT mice were set as 100%). Data are mean ± SEM. ∗*P* < 0.05, ∗∗*P* < 0.01, ∗∗∗*P* < 0.001, ∗∗∗∗*P* < 0.0001. One-way ANOVA in (B), Two-way ANOVA in (F), Student’s t-test in (C-E, G). (For interpretation of the references to colour in this figure legend, the reader is referred to the Web version of this article.)

To elucidate the mechanisms underlying the function of DEL-1 to regulate APC proliferation and BAT adaptation to cold, we next assessed the expression of integrins that could serve as possible DEL-1 receptors in BAT APCs; importantly, different integrins have been previously implicated in regulating BAT thermogenic functions [24,45,46]. Flow cytometric analysis for the integrin β1, β2, β3 and β5 chains on primary APCs isolated from BAT of WT mice, revealed the presence of integrins β1 and β3 on the APC surface (Figure 4A and data not shown). Importantly, the expression of β3 integrin was much more abundant on APCs from BAT than APCs from SAT in cold-exposed mice (Supp. Fig. 5A). Adhesion of BAT-derived primary APCs to immobilized DEL-1 was inhibited by an antibody against β3 integrin but not by an antibody against β1 integrin (Figure 4B). Additionally, primary APC adhesion to DEL-1 was blocked by a cyclic RGDFV peptide with specificity for αvβ3 integrin inhibition (Figure 4C). Thus, DEL-1 interacts with APCs via binding to αvβ3 integrin. Next, we investigated whether DEL-1 directly promotes APC proliferation. Indeed, in the presence of DEL-1, proliferation of primary APCs isolated from BAT (Figure 4D,E) as well as of a brown preadipocyte cell line (Supp. Fig. 5B) was enhanced.

To provide conclusive *in vivo* evidence about the role of the DEL-1/αvβ3 integrin interaction in mediating cold-induced BAT adaptation and APC proliferation, we engaged mice expressing a mutant DEL-1 carrying an Asp-to-Glu substitution in its RGD motif rendering it incapable to interact with RGD-binding integrins, such as αvβ3 integrin (Del1^RGE/RGE^ mice) [29,38]. Cold-challenged Del1^RGE/RGE^ mice became hypothermic compared to their WT control mice (Figure 4F). Furthermore, cold-exposed Del1^RGE/RGE^ mice exhibited reduced numbers of BAT APCs and decreased APC proliferation compared to control mice (Figure 4G). In summary, DEL-1 promotes cold-stimulated BAT function and APC proliferation via its interaction with αvβ3 integrin.

### 2.4 DEL-1 promotes APC proliferation via AKT-driven glycolysis

**Figure 5:**
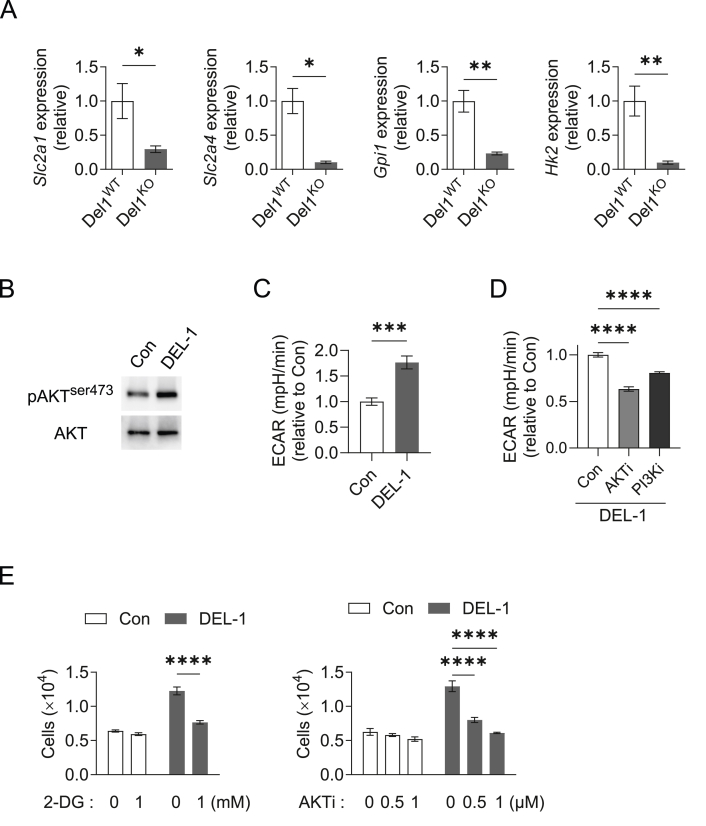
**DEL-1 promotes APC proliferation via AKT-driven glycolysis**. (A) Del1^KO^ and Del1^WT^ mice (*n* = 4/group) were challenged with a cold exposure (temperature of 4 °C for 12 h). APCs were sorted as CD45^−^CD31^−^CD29^+^Sca1^+^PDGFRα^+^ cells from BAT of the mice and expression of glycolysis-related genes was analyzed. 18S expression was used for normalization and gene expression of WT cells was set as 1. *Slc2a1*, solute carrier family 2 member 1; *Slc2a4*, solute carrier family 2 member 4; *Gpi1*, glucose-6-phosphate isomerase 1; *Hk2,* hexokinase 2. (B–E) Primary APCs were sorted as CD45^−^CD31^−^CD29^+^Sca1^+^PDGFRα^+^ cells from BAT of WT mice. (B) Immunoblot analysis of phosphorylated AKT (pAKT^ser473^) and total AKT (AKT) in APCs after treatment without (Con) or DEL-1 for 30 min. Representative blot image from 4 experiments. (C) APCs were pre-incubated without (Con) or with DEL-1 for 30 min and the extracellular acidification rate (ECAR) was analyzed using a Seahorse XFe96 extracellular flux analyzer after addition of glucose. Data are presented as relative to control (Con), set as 1. Representative experiment performed with five replicates; similar results were obtained in three additional experiments. (D) APCs were pre-incubated with DEL-1 in the presence of an inhibitor of AKT (AKTi) or an inhibitor of PI3K (PI3Ki) or DMSO control (Con) for 45 min and ECAR was measured using a Seahorse XFe96 extracellular flux after addition of glucose. Data are presented as relative to the DMSO treated cells (Con), which was set as 1. A representative experiment performed with five replicates is shown; similar results were obtained in two additional experiments. (E) Proliferation of APCs was studied in the absence (Con) or presence of DEL-1, without (0 mM) or with an inhibitor of glycolysis (2-DG) (left), or without (0 μM) or with two different concentrations of an inhibitor of AKT (AKTi) (right). Data are presented as the number of cells after 3 days of culture (*n* = 4 separate cell isolations); the same cell number/well was seeded at the start of the assay, as described in the Materials and Methods. Data are as mean ± SEM. ∗*P* < 0.05, ∗∗*P* < 0.01, ∗∗∗*P* < 0.001, ∗∗∗∗*P* < 0.0001. One-way ANOVA in (D), Two-way ANOVA in (E), Student’s t-test in (A, C) except for *Slc2a4* in panel A (Mann–Whitney *U*-test).

An increase in glycolysis has been implicated as an integral component of cold-stimulated BAT activation and thermogenic recruitment [14]. As we found that DEL-1 deficiency decreased APC proliferation, we next aimed to assess if this effect was linked to alterations in glycolysis. We first addressed whether DEL-1 regulated glycolysis in BAT-derived primary APCs. Primary APCs isolated from the BAT of cold-exposed Del1^KO^ mice displayed decreased expression of glycolysis-related genes, *Slc2a1* (solute carrier family 2 member 1, encoding the glucose transporter GLUT1), *Slc2a4* (solute carrier family 2 member 4, encoding the glucose transporter GLUT4), *Hk2* (encoding the glycolytic enzyme hexokinase 2), *Gpi1* (encoding glucose-6-phosphate isomerase 1), as compared to APCs isolated from Del1^WT^ mice (Figure 5A). AKT signaling has been implicated in brown adipogenesis [47] and promotes glycolysis during BAT thermogenic recruitment [14]. We therefore studied whether DEL-1 regulated APC glycolysis in an AKT-dependent manner. AKT phosphorylation was elevated in APCs isolated from BAT upon treatment with DEL-1 (Figure 5B). Given the major role of AKT signaling in promoting glycolysis of brown adipocytes [14], we next tested whether DEL-1 promotes glycolysis of primary APCs isolated from BAT by performing Seahorse analysis. DEL-1 treatment increased the extracellular acidification rate (ECAR) of BAT-derived primary APCs (Figure 5C). Importantly, the ECAR of DEL-1-treated primary BAT APCs was reduced in the presence of an inhibitor of AKT or PI3K (Figure 5D), indicating that the DEL-1-induced increase in glycolysis was dependent on AKT activity. We finally sought to link the DEL-1-mediated increase in AKT-dependent glycolysis with its effect in promoting APC proliferation. To this end, primary APC proliferation was performed in the absence or presence of DEL-1 without or with inhibitors of glycolysis (2-DG) or an AKT inhibitor. Both inhibitors decreased the DEL-1-stimulated APC proliferation, but failed to affect proliferation in the absence of DEL-1 (Figure 5E). Together, these findings suggest that DEL-1 promotes brown APC proliferation in a manner that requires AKT signaling and glycolysis activation.

## 3 Discussion

**Figure 6:**
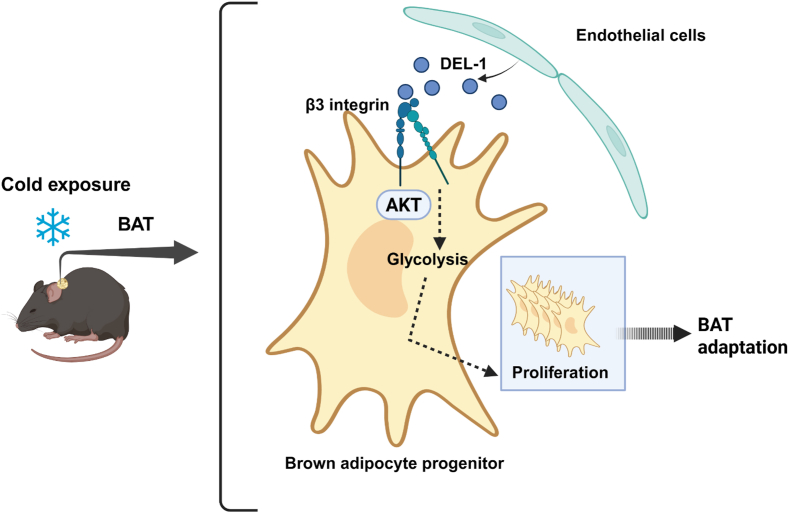
**Graphical Abstract: Endothelial DEL-1 promotes cold-induced BAT APC proliferation and BAT adaptation**. Under cold exposure, the expression of secreted DEL-1 in the vascular endothelium of the BAT is upregulated. DEL-1 interacts with RGD-binding integrins, such as the αvβ3 integrin that is expressed on BAT APCs. The DEL-1/αvβ3 integrin interaction promotes APC proliferation via enhanced AKT signaling and glycolysis activation. This mechanism supports the cold-stimulated BAT adaptation. BAT (brown adipose tissue), DEL-1 (Developmental Endothelial Locus-1). (For interpretation of the references to colour in this figure legend, the reader is referred to the Web version of this article.)

The BAT not only contributes to thermoregulation but also to systemic metabolism and energy homeostasis [2]. The discovery of functional BAT in adult individuals bears important translational implications. Specifically, therapeutic approaches to promote BAT expansion could be used for improving systemic metabolism [6,[48], [49], [50], [51], [52]]. Understanding the complex mechanisms underlying cold-induced BAT activation is therefore of importance. A hallmark of cold-induced BAT adaptation is the rapid and great proliferative response of endothelial cells and perivascular APCs [13,[17], [18], [19]]; however, little was known about the potential crosstalk of these interstitial cell types in cold-stimulated BAT. Our present work has filled this gap by using several genetically modified mouse tools, including DEL-1 deficiency, endothelial cell-exclusive overexpression of DEL-1, and expression of a mutant DEL-1 form that cannot interact with RGD-binding integrins, such as αvβ3 integrin. Our findings establish that DEL-1, secreted from the BAT vessel endothelium, interacts with αvβ3 integrin on APCs and facilitates a local juxtacrine mechanism, which supports cold-stimulated BAT adaptation (Figure 6). AKT signaling-triggered increased glucose uptake and glycolysis is also an integral component of acute BAT adaptation to cold [14]. Importantly, we show that the DEL-1/αvβ3 integrin-dependent juxtacrine endothelial cell/APC crosstalk promoted proliferation of the latter in cold-stimulated BAT via upregulation of AKT signaling and glycolysis (Figure 6). Additionally, expression of glucose transporters and glycolysis-related enzymes was decreased in APCs from BAT from cold-exposed mice owing to DEL-1 deficiency. Hence, our findings suggest that the processes of APC proliferation and increased glycolysis, previously reported as important elements of acute BAT cold acclimation [14,19,53], are functionally and causally interconnected by the herein described DEL-1/αvβ3 integrin-dependent juxtacrine endothelial cell/APC crosstalk.

The DEL-1 upregulation we observed in the BAT upon cold exposure could in principle result from the enhanced formation of new vessels of the tissue [17]; however, this may only partially explain DEL-1 upregulation since we also showed enhanced mRNA expression of DEL-1 in sorted endothelial cells from the BAT of cold-exposed mice. We also tested the effects of factors that stimulate APC proliferation, such as the sympathoadrenergic agonist NE or the thyroid hormone T3 [13,54], on endothelial DEL-1 expression. Interestingly, T3 but not NE treatment significantly upregulated DEL-1 expression. Consistently, no upregulation of DEL-1 expression in the BAT of mice was observed upon treatment with the β3 adrenergic receptor agonist CL316243 [40]. In addition, DEL-1 deficiency had no impact on the thermogenic BAT response caused by CL316243 (data not shown). The role of endothelium-derived DEL-1 in BAT cold adaptation was further confirmed by using mice with endothelial-specific overexpression of DEL-1 and global DEL-1 deficiency (EC-Del1/Del1^KO^); in these mice the impaired cold tolerance and BAT adaptation observed in Del1^KO^ mice were reversed. However, due to the transgenic overexpression, EC-Del1/Del1^KO^ mice displayed higher *Edil3* expression in their BAT compared to that of wild-type mice. Nevertheless, as these mice express DEL-1 exclusively in the endothelium, we conclude that endothelial-derived DEL-1 promotes BAT cold adaptation. Future studies should aim to elucidate the molecular mechanisms underlying the cold- and/or T3-induced transcriptional upregulation of DEL-1 in BAT endothelial cells.

Compared to BAT, DEL-1 expression in white adipose tissue depots (SAT and VAT), was much lower and not stimulated by cold challenge. In addition, while β3 integrin was present on APCs isolated from BAT, its expression on SAT-derived APCs was rather negligible. Consistently, DEL-1 deficiency reduced APC proliferation in the BAT but not in the SAT of cold-exposed mice. Interestingly, αvβ5 integrin, which is closely related to αvβ3 integrin, promotes cold-induced beige adipogenesis in the white adipose tissue, via binding to irisin [24,39]. In turn, we found β5 integrin to be absent from BAT APCs. Our data together with these previous findings allow us to formulate the hypothesis that functionally similar, yet distinct, αv integrin-dependent interactions are operative during cold-triggered BAT neogenesis and beige adipogenesis in the white adipose tissue: While the DEL-1/αvβ3 integrin-dependent interaction contributes to APC proliferation in BAT and BAT cold adaptation, the irisin/αvβ5 integrin-dependent binding promotes cold-induced beige adipogenesis in the white fat [24,39].

Upregulation of UCP-1 is a major component of BAT thermogenic recruitment in cold [2]. Recently, two distinct cell subsets were discovered in the BAT of mice, which include, besides the high-thermogenic, classical brown adipocytes, also a low-thermogenic cell population with lower UCP-1 expression [55]. Upon cold challenge the latter population is activated to give rise to high-thermogenic brown adipocytes. It would be tempting to assess in the future whether the herein described DEL-1/αvβ3 integrin-dependent crosstalk promoting APC proliferation results in generation of low- or high-thermogenic adipocytes.

Modulation of BAT to promote its expansion has garnered increasing attention recently as a therapeutic strategy for improving systemic metabolism [6,[48], [49], [50], [51], [52]]. A limitation of our study is that we did not study the role of DEL-1 in the context of diet-induced obesity. A comprehensive investigation into the effects of DEL-1 in obesity and related metabolic dysregulation as well as potential underlying mechanisms warrants a dedicated future study. Notwithstanding this and other open questions, our findings suggest that the DEL-1/αvβ3 integrin interaction, through its role in BAT acclimation to cold, may hold therapeutic potential. Enhancing this interaction to promote APC proliferation could support BAT function in diverse metabolic contexts, a hypothesis that merits further evaluation in preclinical animal models.

## 4 Materials and methods

### 4.1 Animal studies

Del1^KO^ mice have been previously described [28]. For generating mice with exclusive endothelial-specific overexpression of DEL-1 and lacking DEL-1 in all other cell types (EC-Del1/Del1^KO^), we crossed the previously described endothelial-specific DEL-1 overexpressing mice (EC-Del1, generated by utilizing a Tie2 promoter/enhancer construct) [36,43,44] with DEL-1-deficient mice. Del-1^RGE/RGE^ mice that express an RGE point mutant isoform of DEL-1, making it incapable of interacting with αvβ3 integrin, created by one-step CRISPR/Cas-mediated genome editing, were also previously described [29,38]. Wild-type mice (C57BL/6) were also purchased from Charles River. Mice were housed on a standard 12 h light/12 h dark cycle with food and water supplied ad libitum under specific pathogen-free conditions. In acute cold exposure experiments, mice were challenged by exposure to a temperature of 4 °C, being individually housed in pre-chilled cages for 12 h with free access to food and water. To streamline animal experiments performed at two different locations (Technische Universität Dresden, Germany and the University of Pennsylvania, USA), in several experiments (as indicated in the figure legends), mice were fed with the same normal diet (D12450B, Research Diets) for 8 weeks and the acute cold exposure experiments were performed thereafter as described above. In other experiments, mice received the β3-adrenergic receptor agonist CL316243 (1 mg/kg, #1499, Tocris Bioscience) intraperitoneally (i.p.), or PBS as control, for 4 days and euthanized 4 h after the last injection. Animal experiments were approved by the Landesdirektion Sachsen, Germany, or the Institutional Animal Care and Use Committee of the University of Pennsylvania.

### 4.2 Isolation, culture and proliferation of primary adipocyte progenitor cells (APCs) from the brown adipose tissue (BAT)

Mouse primary APCs were isolated from interscapular BAT. BAT from C57BL/6 mice was minced and digested with collagenase I (1 mg/ml, #17100-017, Gibco) in DMEM (high glucose, #31966, Gibco) containing 0.5% bovine serum albumin (BSA, #A7030, Sigma Aldrich) for 60 min at 37 °C with shaking. The digested tissue was then filtered through a 100 μm cell strainer and centrifuged at 600 g for 10 min. The isolated stromal vascular fraction (SVF) was incubated with 1x RBC lysis buffer (#00-4300-54, Invitrogen) for 5 min at room temperature and then filtered through a 40 μm cell strainer. The washed SVF cells were resuspended with PBS containing 0.5% BSA and 2 mM EDTA and stained with anti-CD31 (clone MEC 13.3, #553373, BD), anti-CD45 (clone 30-F11, #553081, BD), anti-CD29 (clone HMβ1-1, #102226, Biolegend), anti-Sca1 (clone D7, #558162, BD), and anti-PDGFRα (clone APA5, #562774, BD) for 20 min at 4 °C after incubation with mouse Fc Block (clone 2.4G2, #553141, BD). The SVF cells were washed and APCs were sorted as CD31^−^CD45^−^CD29^+^Sca1^+^PDGFRα^+^ cells by using a FACSAria II cell sorter (BD). The sorted progenitors were cultured in DMEM/F12 medium supplemented with 10% FBS, glutamax, and penicillin/streptomycin until reaching 70–80% confluency. For performing cell proliferation assay, the cells were harvested utilizing a non-enzymatic cell dissociation solution (Sigma); the same cell number (3,000 cells/well) was seeded into a 48-well plate with complete DMEM/F12 medium. On the next day, cells were treated with recombinant human DEL-1 protein (EDIL3, 0.5 μg/ml, #6046-ED-050, R&D Systems) or PBS. In some experiments, cells were also treated with the glycolysis inhibitor 2-Deoxy-d-glucose (2DG, 1 mM, #D8375, Sigma) or the AKT1/AKT2 inhibitor Akti-1/2 (0.5 μM or 1 μM, #S7776, Selleckchem). After 3 days, cells were harvested and counted with a hemocytometer. For Ki67 analysis, cells were harvested after 2 days of culturing and incubated with anti-Ki67 antibody (clone SolA15, #11-5698-82, eBioscience) after fixation/permeabilization with FOXP3 staining kit (#00-5523-00, eBioscience) and analyzed by flow cytometry (BD FACSCanto™ II), as described below.

### 4.3 BAT endothelial cell isolation, culture and treatment

The SVF cells from BAT of mice were isolated as described in the previous paragraph and endothelial cells were isolated by CD146 positive selection (#130-092-007, Milteny Biotec) with a LS column. Isolated CD146-positive cells were pooled from 2 mice for each replicate and cultured in a 6-well plate with endothelial cell growth medium (#C-22010, Promo Cell) and SupplementMix (#c-39215, Promo Cell). After 6 days, cells were seeded into a 12-well plate and on the next day they were treated with 3,3′,5-Triiodo-l-thyronine (T3, 10 nM, #T2877, Sigma) or l-Norepinephrine hydrochloride (NE, 1 μM, #74480, Sigma) for 12 h; thereafter the cells were harvested for RNA isolation and gene expression analysis.

### 4.4 Immortalized brown preadipocyte cell proliferation assay

Brown preadipocyte cell line [[56], [57], [58], [59]] was maintained in DMEM (high glucose, #31966, Gibco) containing 20% FBS and penicillin/streptomycin. For cell proliferation assay, cells were harvested by using non-enzymatic cell dissociation solution (Sigma) and 3,000 cells/well were seeded into a 48-well plate with complete DMEM medium containing 10% FBS. On the next day, cells were treated with recombinant human DEL-1 protein (EDIL3, 0.5 μg/ml, #6046-ED-050, R&D Systems) or PBS. After 3 days, cells were harvested and counted with a hemocytometer.

### 4.5 Flow cytometry and sorting

The SVF cells from BAT or SAT of mice were isolated as described in the paragraph “Isolation, culture and proliferation of primary adipocyte progenitor cells (APCs) from the brown adipose tissue (BAT)” and resuspended with PBS containing 0.5% BSA and 2 mM EDTA. Cells were then incubated with mouse Fc Block (clone 2.4G2, #553141, BD) and stained with anti-CD31 (clone MEC 13.3, #553373, BD), anti-CD45 (clone 30-F11, #553081, BD), anti-CD29 (clone HMβ1-1, #102226, Biolegend), anti-Sca1 (clone D7, #558162, BD), and anti-PDGFRα (clone APA5, #562777, BD) for 20 min at 4 °C. After washing, cells were fixed and permeabilized by using the FOXP3 staining kit (#00-5523-00, eBioscience) and stained with anti-Ki67 (clone SolA15, #11-5698-82, eBioscience) for 30 min at 4 °C. Stained cells were washed and analysed using a BD FACSCanto™ II or Novocyte flow cytometer (ACEA Biosciences) and data were analyzed by FlowJo software or NovoExpress software, respectively. APCs were defined as CD31^−^CD45^−^CD29^+^Sca1^+^PDGFRα^+^ cells and proliferating cells were determined as Ki67 positive cells.

For gene expression analysis in APCs or endothelial cells, BAT SVF cells were isolated as described under “Isolation, culture and proliferation of primary adipocyte progenitor cells (APCs) from the brown adipose tissue (BAT)” and were stained with anti-CD31 (clone 390, #7-0311-82, eBioscience), anti-CD45 (clone 104, #11-0454-82, eBioscience), anti-CD29 (clone HMβ1-1, #102226, Biolegend), anti-Sca1 (clone D7, #558162, BD), and anti-PDGFRα (clone APA5, #562774, BD) for 20 min at 4 °C after incubation with mouse Fc Block (clone 2.4G2, #553141, BD). Endothelial cells were sorted as CD31^+^CD45^-^ and APCs were sorted as CD31^−^CD45^−^CD29^+^Sca1^+^PDGFRα^+^ cells by using a FACSAria II cell sorter (BD).

For integrin expression analysis, isolated APCs were cultured in DMEM/F12 medium supplemented with 10% FBS, glutamax, and penicillin/streptomycin until reaching 70–80% confluency and were then stained with antibodies against β1 integrin (CD29, clone HMβ1-1, #102226, Biolegend) or β3 integrin (CD61, clone 2C9.G3, #11-0611-82, eBioscience) and analyzed by flow cytometry (BD FACSCanto™ II). Isotype control antibodies, APC-Cy7 Armenian Hamster IgG Isotype (clone HTK888, #400927, Biolegend), or FITC Armenian Hamster IgG Isotype (clone eBio299Arm, #11-4888-81, eBioscience) were used as a negative control. The histogram of β1 or β3 integrin expression with appropriate isotype control was analyzed by using the FlowJo software.

### 4.6 Cell adhesion assays

Adhesion assay was performed as previously described with some modifications [27]. Briefly, a Nunc MaxiSorp™ Flat Bottom 96 well plate (#442404, Thermo Fisher Scientific) was coated with recombinant human DEL-1 protein (10 μg/ml, R&D Systems) overnight at 4 °C and the plate was then blocked with 3% BSA in PBS for 1 h at room temperature after washing twice with PBS. Primary BAT-derived APCs, isolated as described under “Isolation, culture and proliferation of primary adipocyte progenitor cells (APCs) from the brown adipose tissue (BAT)”, were harvested utilizing a non-enzymatic cell dissociation solution (Sigma) from the cell culture plate and cells were incubated with BCECF-AM (1 μM, #B1170, Invitrogen) for 20 min at 37 °C. After washing, cells (2 × 10^4^) were resuspended in HBSS (free of Ca and Mg) including 0.1% BSA and incubated with cyclic RGDFV (10 μM, # SCP0111, Sigma), a peptide antagonist with specificity for αvβ3 integrin, for 20 min at room temperature and then added into DEL-1-coated wells in HBSS containing 0.5 mM Mn^2+^ and incubated for 20 min at 37 °C. For performing antibody-mediated blockade, primary APCs (2 × 10^4^ cells) were pre-incubated with anti-β1 integrin (10 μg/ml, clone HMβ1-1, #102210, Biolegend), anti-β3 integrin (10 μg/ml, clone HMβ3-1, #104310, Biolegend), or Armenian hamster IgG (10 μg/ml, clone HTK888, #400916, Biolegend) as control for 20 min at 4 °C and then added into DEL-1-coated wells in HBSS containing 0.5 mM Mn^2+^ and incubated for 20 min at 37 °C.

In all adhesion experiments, fluorescence intensity was measured after the incubation time, before and after the washings, by using a Synergy HT multi-mode microplate reader (Biotek Instruments), as a readout of the total added cells and the finally adherent cells, respectively. The percentage of adherent cells was calculated as (fluorescence intensity of adherent cells/fluorescence intensity of input cells) × 100.

### 4.7 Glycolysis assay

Primary APCs from BAT were isolated and cultured as described under “Isolation, culture and proliferation of primary adipocyte progenitor cells (APCs) from the brown adipose tissue (BAT)” and the glycolytic rate assay was analyzed in a XF96 Extracellular Flux Analyzer (Seahorse Bioscience). Briefly, cells (0.5 or 1 × 10^4^/well) in Seahorse basal DMEM medium supplemented with 1 mM glutamine were incubated with recombinant human DEL-1 protein (0.5 μg/ml, R&D Systems) or PBS. For inhibitors treatment, cells were treated with the AKT inhibitor, Akti-1/2 (2 μM, Selleckchem) or the PI3Kα/δ/β inhibitor Ly294002 (10 μM, #L9908, Sigma) in the presence of DEL-1 for 45 min at 37 °C in a non-CO_2_ incubator. Glycolytic rate assay was performed by sequentially adding glucose (10 mM), oligomycin (1 μM), and 2-Deoxy-d-glucose (2DG, 50 mM) according to the Seahorse XF glycolysis stress test kit (#103020-100, Agilent). Extracellular acidification rate (ECAR) was acquired after normalization of cell numbers by DNA measurement with CyQUANT™ (#C7026, Thermo Fisher Scientific). Glycolysis was calculated as “(maximum rate measurement after glucose and before oligomycin injection) – (last rate measurement before glucose injection)”, following the manufacturer’s manual.

### 4.8 ELISA and immunoblotting

BAT tissues were snap frozen in liquid nitrogen and homogenized by Precellys 24 tissue homogenizer (Bertin instruments) in lysis buffer (150 mM NaCl, 1% Triton X-100, 0.5% sodium deoxycholate, 0.1% SDS, 50 mM Tris–HCl, pH 7.5) containing a protease and phosphatase inhibitor cocktail (#11697498001, # 4906845001, Roche) and then centrifuged at 13,000 g for 20 min at 4 °C. The supernatant was collected and total proteins were quantified by a BCA protein assay kit (#23227, Thermo Fisher Scientific). For UCP-1 quantification, BAT lysate samples containing 1 μg protein were used and analyzed with a mouse UCP-1 ELISA kit (#A303388, antibodies.com).

For AKT immunoblotting, primary APCs isolated from BAT (4 × 10^4^ cells), as described under “Isolation, culture and proliferation of primary adipocyte progenitor cells (APCs) from the brown adipose tissue (BAT)” were treated with recombinant human DEL-1 protein (0.5 μg/ml, R&D Systems) or PBS for 30 min and cells were lysed. Denatured samples (10 μg) were separated on SDS-PAGE gels and transferred to PVDF membrane and incubated with primary antibody against phospho-AKT (Ser473, #4060, Cell Signaling) and subsequently, after stripping the membrane by using Restore™ Western Blot Stripping-Buffer (#21059, ThermoFisher Scientific), with antibody against AKT (#9272, Cell Signaling). Upon incubation with appropriate secondary antibodies, the proteins were detected by using the SuperSignal West Pico Chemiluminescent substrate (#34579, Thermo Fisher Scientific).

### 4.9 Immunohistochemistry

Isolated BAT samples were fixed in 4% PFA solution for 24 h, embedded in paraffin and cut into 5 μm sections. For UCP-1 immunohistochemistry, sections were deparaffinised and incubated with citrate buffer and then incubated with an antibody against UCP-1 (#ab10983, Abcam) overnight at 4 °C. The Vectastain ABC kit (#PK-4001, Vector Laboratories) was used for UCP-1 detection. Images were acquired by utilizing a ZEISS Axio Observer Z1-computerized microscope.

### 4.10 Gene expression analysis

White fat tissues (SAT, VAT) and BAT were snap frozen in liquid nitrogen and homogenized by using the Precellys 24 tissue homogenizer (Bertin Instruments) in Trizol (#TR118, MRC). APCs were sorted from BAT of mice, sorted as described under “Flow cytometry and sorting” using a FACSAria II cell sorter (BD), and were lysed in Trizol. Isolated RNA was reverse-transcribed with the iScript cDNA Synthesis Kit (#1708891, Bio-Rad) and qPCR was performed by using the SsoFast EvaGreen Supermix (#1725201, BioRad) and gene-specific primers in a CFX384 Real-time PCR (BioRad). Relative mRNA expression levels were calculated according to the ^ΔΔ^Ct method upon normalization to 18S or *Actb*. The mouse primer sequences (5'-> 3′) used in this study are:

*Edil3*, F: CCTGTGAGATAAGCGAAGC, R: GAGCTCGGTGAGTAGATG
*Slc2a1*, F: CATTGTGGCCGAGCTGTTC, R: CGCACAGTTGCTCCACATAC
*Slc2a4*, F: CAGCTCAGCTAGTGCGTCAG, R: GATTCTGCTGCCCTTCTGTC
*Gpi1*, F: AATCGCCTCCAAGACCTTCA, R: CGAGAAACCACTCCTTTGCTGT
*Hk2*, F: CCGCCGTGGTGGACAAGATA, R: AGCAGTGATGAGAGCCGCTC
*Ccna2*, F: CAGCATGAGGGCCATCCTT, R: GCAGGGTCTCATTCTGTAGTTTATATTCT
*Ccnd2*, F: CACGACTTCATTGAGCACATCCT, R: GCGGATCAGGGACAGCTTCT
*Ccng1*, F: GCGACTGAAGAGGAAAGGAATGT, R: TGAAACCGTGAACCTATACTGACTT
*Actb*, F: CGTGGGCCGCCCTAGGCACCA, R: TTGGCCTTAGGGTTCAGGGGGG
18S, F: GTTCCGACCATAAACGATGCC, R: TGGTGGTGCCCTTCCGTCAAT

### 4.11 Statistical analysis

For statistical comparisons of two groups of samples, a two-tailed Student’s t-test was used for values that followed normal distribution, as assessed by the Shapiro–Wilk normality test. For non-normally distributed data, a Mann–Whitney *U* test was performed. For comparisons of more than two groups, ordinary one-way analysis of variance (ANOVA) or two-way ANOVA followed by Bonferroni multiple comparisons test was used. Statistical analyses were performed using GraphPad Prism (v10.2.3) and significance was set at *p* < 0.05.

## CRediT authorship contribution statement

**Kyoung-Jin Chung:** Conceptualization, Formal analysis, Investigation, Methodology, Visualization, Writing – original draft, Writing – review & editing. **Antonios Chatzigeorgiou:** Writing – original draft. **Jong-Hyung Lim:** Formal analysis, Investigation. **Xiaofei Li:** Formal analysis, Investigation. **Ismini Marava:** Investigation. **Dong-Young Kim:** Investigation. **Anke Witt:** Investigation. **Janine Gebler:** Investigation. **Sylvia Grossklaus:** Investigation, Resources. **Bettina Gercken:** Investigation. **Irakli Kopaliani:** Resources. **Pallavi Subramanian:** Resources. **Matthias Blüher:** Resources. **Khalil Bdeir:** Resources. **Vasileia Ismini Alexaki:** Methodology, Project administration, Writing – review & editing. **George Hajishengallis:** Funding acquisition, Project administration, Supervision, Writing – review & editing. **Triantafyllos Chavakis:** Conceptualization, Funding acquisition, Project administration, Supervision, Writing – original draft, Writing – review & editing.

## Declaration of competing interest

The authors declare the following financial interests/personal relationships which may be considered as potential competing interests: Matthias Bluher reports a relationship with Abbott, Amgen, 10.13039/100004325AstraZeneca, 10.13039/100004326Bayer, Boehringer Ingelheim, 10.13039/501100002973Daiichi-Sankyo, 10.13039/100004312Lilly, 10.13039/100030732MSD, 10.13039/501100004191Novo Nordisk, 10.13039/100004339Sanofi that includes: consulting or advisory and speaking and lecture fees. If there are other authors, they declare that they have no known competing financial interests or personal relationships that could have appeared to influence the work reported in this paper.

## Data Availability

Data will be made available on request.
