# Supplementary material for: A DEL-1/αvβ3 integrin axis promotes brown adipocyte progenitor proliferation and cold-induced brown adipose tissue adaptation
Source: Mol Metab. 2025 Aug 7;100:102229. doi: 10.1016/j.molmet.2025.102229 (PMC12391742; doi:10.1016/j.molmet.2025.102229)
Supplement: Multimedia component 1 [file mmc1.pdf]

Supp figure 1

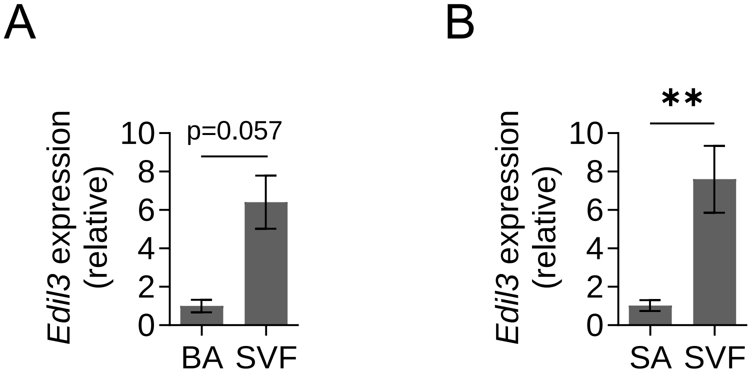

Supp figure 2

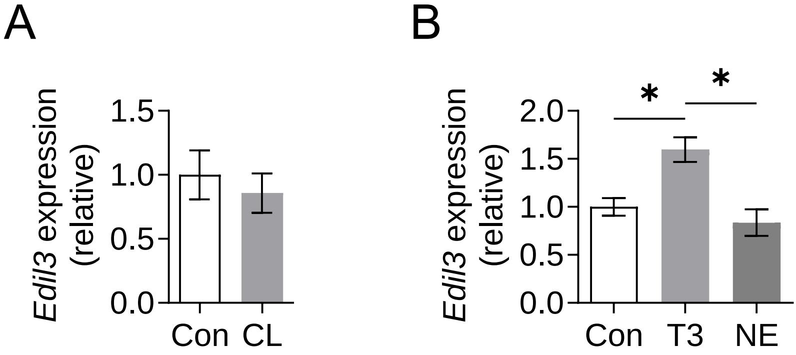

Supp figure 3

A

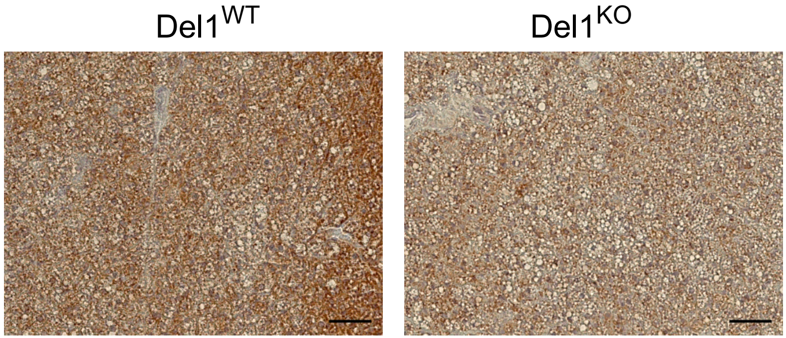

B

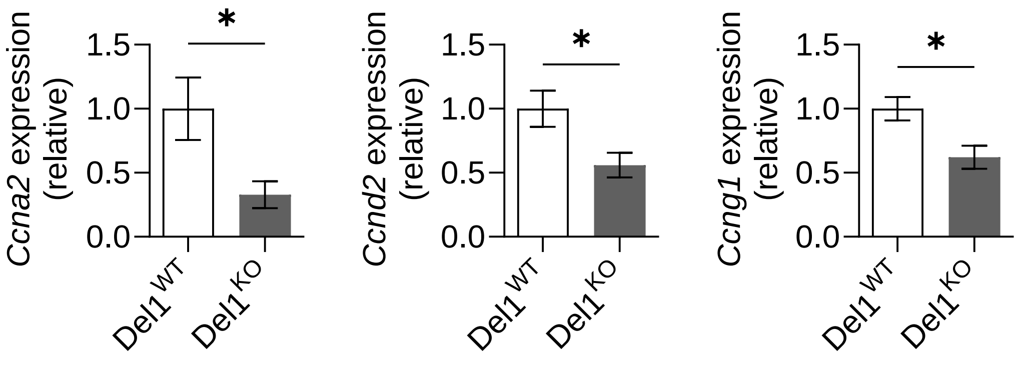

Supp figure 4

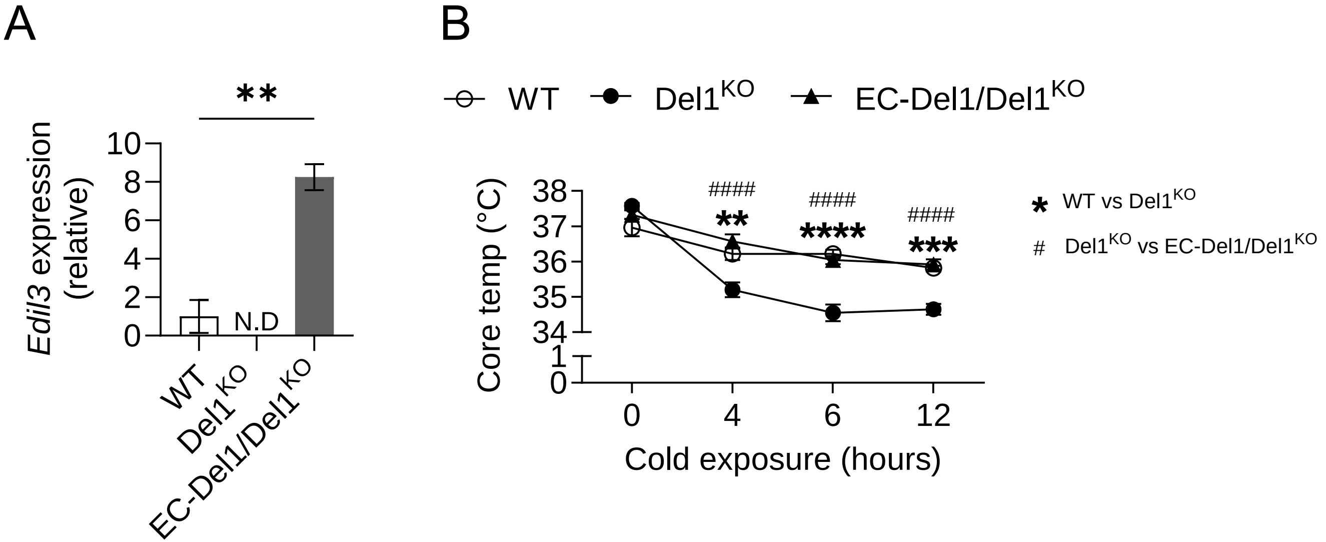

Supp figure 5

A

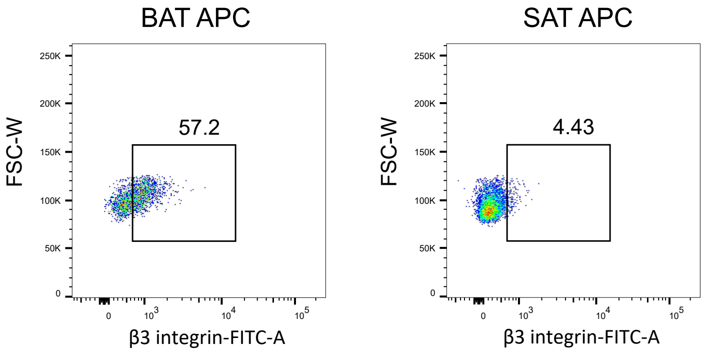

B

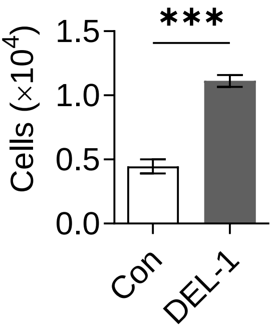

SUPPLEMENTARY FIGURE LEGENDS

Suppl. Figure 1. DEL-1 is mainly expressed in the SVF of different adipose tissues.

(A) *Edil3* mRNA expression in the adipocyte fraction containing brown adipocytes (BA) or the stromal vascular fraction (SVF) from the BAT, and (B) *Edil3* mRNA expression in the adipocyte fraction (SA) or the stromal vascular fraction (SVF) from the subcutaneous white adipose tissue from mice at room temperature (n=3-4). 18S expression was used for normalization and *Edil3* expression of BA or SA was set as 1. Data are mean ± SEM. **P < 0.01. Mann-Whitney *U*-test in (A), Student's t-test in (B).

Suppl. Figure 2. Regulation of DEL-1 expression in endothelial cells of the BAT.

(A) Wild-type mice received the β3-adrenergic receptor agonist CL316243 (CL) or PBS (Con). *Edil3* mRNA expression in the BAT is shown (n=6 mice per group). 18S expression was used for normalization and *Edil3* expression of the PBS control group was set as 1. (B) Endothelial cells were isolated from BAT of wild-type mice and cultured cells were treated with 3,3′,5-Triiodo-L-thyronine (T3, 10 nM) or L-Norepinephrine hydrochloride (NE, 1 μM) or PBS as control (Con), as described in the Materials and Methods. *Edil3* expression was analyzed; 18S was used for normalization and gene expression of control-treated cells was set as 1 (n=3). Data are mean ± SEM. *P < 0.05. One-way ANOVA in (B).

Suppl. Figure 3. DEL-1 promotes thermogenic adaptation and APC proliferation in cold-stimulated BAT.

$Del1^{KO}$ and $Del1^{WT}$ mice were challenged with cold exposure (temperature of 4°C for 12 hours). (A) Representative images of UCP-1 staining from BAT of $Del1^{WT}$ or $Del1^{KO}$ mice. Scale bar, 50μm. (B) APCs were sorted as $CD45^{-}CD31^{-}CD29^{+}Sca1^{+}PDGFR\alpha^{+}$ cells from the BAT of $Del1^{WT}$ or $Del1^{KO}$ mice (n=5-6) and expression of cell cycle-related genes was analyzed. 18S was used for normalization and gene expression of $Del1^{WT}$ cells was set as 1. *Ccna2*, cyclin A2; *Ccng1*, cyclin G1; *Ccnd2*, cyclin D2. Data are mean ± SEM. *P < 0.05. Mann-Whitney *U*-test in (B) except for *Ccnd2* (Student's t-test).

Suppl. Figure 4. DEL-1 expression analysis.

Wild-type (WT; n=5), $Del1^{KO}$ (n=4), and EC-Del1/$Del1^{KO}$ (n=7) mice were challenged with a cold exposure (temperature of 4°C) and *Edil3* mRNA expression in the interscapular BAT was analyzed. 18S expression was used for normalization and *Edil3* expression of BAT from WT mice was set as 1. N.D., not detected. (B) Core body temperature during the cold exposure. Data are mean ± SEM. **P < 0.01, ***P < 0.001, ****P < 0.0001, $^{####}$P < 0.0001. Mann-Whitney *U*-test in (A), Two-way ANOVA in (B).

Suppl. Figure 5. Expression of β3 integrin on APCs and DEL-1 promotes proliferation of brown preadipocytes.

(A) Wild-type mice (n=5) were challenged with a cold exposure (temperature of 4°C for 12 hours) and β3 integrin expression in primary APCs (defined as $CD45^-CD31^-CD29^+Sca1^+PDGFR\alpha^+$ cells) from the BAT or SAT was analyzed by flow cytometry. Representative flow cytometry plots showing the percentage of β3 integrin-positive cells from APCs of BAT or SAT. (B) Proliferation of a brown preadipocyte cell line was studied in the absence (Con) or presence of DEL-1. Data are presented as the number of cells after 3 days of culture (n=3 independent experiments); the same cell number/well was seeded at the start of the assay, as described in the Materials and Methods. Data are mean ± SEM. ***P < 0.001. Student's t-test in (B).
